# Nomogram for predicting preoperative regional lymph nodes metastasis in patients with metaplastic breast cancer: a SEER population-based study

**DOI:** 10.1186/s12885-021-08313-6

**Published:** 2021-05-17

**Authors:** Mi Zhang, Biyuan Wang, Na Liu, Hui Wang, Juan Zhang, Lei Wu, Andi Zhao, Le Wang, Xiaoai Zhao, Jin Yang

**Affiliations:** Department of Medical Oncology, The First Affiliated Hospital of Xi’an Jiaotong University, No 277 Yanta West Road, Xi’an, Shaanxi 710061 People’s Republic of China

**Keywords:** Breast cancer, Metaplastic breast cancer, Lymph nodes metastasis, SEER, Nomogram

## Abstract

**Background:**

Metaplastic breast cancer (MBC) is a rare subtype of breast cancer, and generally associated with poor outcomes. Lymph nodes metastasis (LNM) is confirmed as a critical independent prognostic factor and determine the optimal treatment strategies in MBC patients. We aimed to develop and validate a nomogram to predict the possibility of preoperative regional LNM in MBC patients.

**Methods:**

MBC patients diagnosed between 1990 and 2016 in the Surveillance, Epidemiology, and End Results (SEER) database were included and stochastically divided into a training set and validation set at a ratio of 7:3. The risk variables of regional LNM in the training set were determined by univariate and multivariate logistic regression analyses. And then we integrated those risk factors to construct the nomogram. The prediction nomogram was further verified in the verification set. The discrimination, calibration and clinical utility of the nomogram were evaluated by the area under the receiver operating characteristic (ROC) curve (AUC), calibration plots and decision curve analysis (DCA), respectively.

**Results:**

A total of 2205 female MBC patients were included in the study. Among the 2205 patients, 24.8% (546/2205) had positive regional lymph nodes. The nomogram for predicting the risk of regional LNM contained predictors of grade, estrogen receptor (ER) status and tumor size, with AUC of 0.683 (95% confidence interval (CI): 0.653–0.713) and 0.667 (95% CI: 0.621–0.712) in the training and validation sets, respectively. Calibration plots showed perfect agreement between actual and predicted regional LNM risks. At the same time, DCA of the nomogram demonstrated good clinical utilities.

**Conclusions:**

The nomogram established in this study showed excellent prediction ability, and could be used to preoperatively estimate the regional LNM risk in MBC.

## Background

Metaplastic breast cancer (MBC) is a rare neoplasm, accounting for approximately 0.02–5% of breast cancer (BC) [1, 2]. It is characterized by the presence of two or more components in histology, usually representing a mixture of epithelial (e.g., adenocarcinoma) and mesenchymal (e.g., matrix, spindle cell, and sarcomatous) components [3, 4]. MBC was first described by Huvos et al. in 1973 [5], and considered to be a distinct histologic subtype in the 2000 World Health Organization (WHO) guidelines for histologic classification of tumors of the breast.

MBC is associated with poor prognosis, and lymph nodes metastasis (LNM) is an important prognostic determinant for patients with BC. Accurately preoperative evaluation of regional LNM is critical for determining the optimal treatment strategies for MBC patients. Neoadjuvant systemic therapy (NAST) has many potential advantages, including: downstaging the breast cancer and axilla, improving prognostication based on response and the chances of breast-conserving surgery. Hence, it is increasingly used in patients with clinically node-positive BC [6]. Moreover, patients with extensive axillary nodal involvement planned for irradiation of regional lymph nodes may benefit from reducing the probability of locoregional recurrence [7, 8]. Currently, sentinel lymph node biopsy (SLNB) is performed in all clinically node-negative patients to probe axillary lymph node status. And modified radical mastectomy with axillary lymph node dissection (ALND) remains one of the most effective surgical methods for patients with local advanced BC without distant metastasis.

However, different from other types of BC, the incidence of LNM in MBC is low. The largest study showed the incidence of axillary lymph nodes (ALNs) involvement in MBC was 21.9%, significantly lower than 34.3% in infiltrating ductal carcinoma (IDC) [1]. Wargotz et al. also found ALNs metastasis rate in MBC ranged from 6 to 26% [9]. In addition, by reviewing the data from the previous study, we found that sentinel LNM occurred in only 30% of BC patients with clinically negative lymph nodes. And SLNB had an inherent false-negative rate of 5–10% [10, 11]. In many BC patients, the histopathological examination of their dissected ALNs revealed no metastasis [12]. Furthermore, owing to the advances in treatment, the overall breast cancer death rate has decreased rapidly [13]. In this scenario, health-related quality of life has become more and more important. But, SLNB and ALND are both associated with the risk of the complications, including breast cancer related lymphedema (BCRL) [14], axillary web syndrome [15], numbness, paraesthesia [10, 16], reduced range of motion, upper limb pain [17–19], cancer-related fatigue [20, 21] and so on. Notably, approximately 20% of breast cancer survivors (BCS) will develop BCRL, which is a lifelong threat due to its protracted time of onset [22]. And cancer-related fatigue is also a very common long-term side effect in BCS. A previous meta-analysis involving 12,327 BCS indicated about a quarter of BCS suffered from severe fatigue [23]. Health-related quality of life and psychological health are severely impaired in patients with BC [24, 25]. Based on the above reasons, MBC without LNM may not require additional SLNB and ALND to avoid overtreatment. Therefore, adequate and accurate assessment and prediction of preoperative regional lymph node status are very important. If we can predict lymph node status before surgery, lymph node negative patients could avoid unnecessary treatment.

At present, mammography, ultrasonography, computed tomography, and magnetic resonance are the main methods used to evaluate lymph node status [26–29]. Nevertheless, it is insufficient to screen and evaluate the lymph node status of MBC patients based solely on the imaging appearance. More importantly, MBC is difficult to identify preoperatively on biopsy [30, 31]. Recently, nomogram for predicting the possibility of preoperative LNM has been proven effective and widely used. Unfortunately, so far, there is no nomogram to predict preoperative regional LNM in patients with MBC. Hence, we retrospectively analyzed the clinical characteristics of a large cohort of female MBC patients, aiming to establish an easy, reliable and sensitive clinical risk factor model to predict the risk of regional LNM before surgery.

## Methods

### Patient selection and data collection

The retrospective study was based on the SEER program. The SEER database is an open access resource for cancer-based clinical data, and no ethics committee review approval was needed. We included patients diagnosed with microscopically confirmed MBC between 1990 and 2016. Only patients with MBC as their only cancer were included. The ICD-O-3 codes included in this study were 8052, 8070–8072, 8074, 8560, 8571, 8572, 8575, and 8980, based on previously published studies [32, 33]. The following clinicopathological factors were extracted from the SEER database: age at diagnosis, gender, race, marital status, grade, laterality, estrogen receptor (ER) status, progesterone receptor (PR) status, human epidermal growth factor receptor-2 (HER-2) status, tumor size and regional lymph node status. We excluded male patients and patients with unknown regional lymph node status, laterality, ER status, PR status, race, tumor size, stage and grade.

### Statistical analysis

Chi-square test was used to compare categorical variables between the training set and the validation set. Univariable and multivariable binary logistic regression analyses were utilized to identify factors associated with regional LNM. Variables with *P* < 0.05 were included in the nomogram. The accuracy of the nomogram was evaluated by the discrimination and calibration ability. Discrimination was validated by the area under the receiver operating characteristic (ROC) curve (AUC). Calibration (visualized as the calibration plot) was used to illustrate the correlation between the actual probability and the predicted probability of regional LNM. Clinical usefulness was estimated with decision curve analysis (DCA). Analyses were conducted by SPSS (version 18.0; SPSS, Inc., Chicago, IL) and the packages (rms, hmisc, rmda, etc.) in R software version 4.0.2 (http://www.r-project.org). A two-sided *P*-value less than 0.05 was considered statistically significant.

## Results

### Patient characteristics

**Fig. 1:**
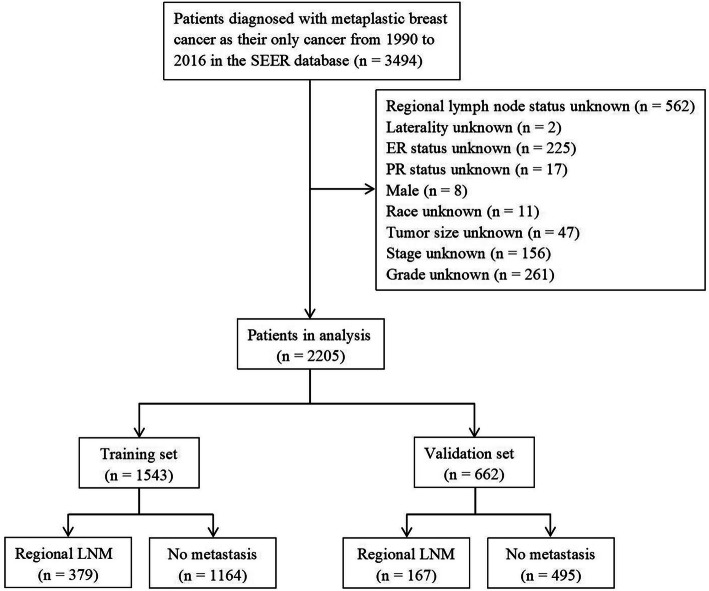
Patient enrollment and exclusion process in the SEER database

In this study, we included 2205 female MBC patients diagnosed from 1990 to 2016. The flow diagram for concrete steps to patient selection is shown in Fig. 1. Table 1 summarized the clinicopathological features of 2205 female MBC patients in detail. Majority patients were married white women. 81.8% (1803/2205) were poorly differentiated or undifferentiated. 74.1% (1633/2205) of patients were diagnosed with a tumor larger than 2 cm. Most of the tumors lacked ER, PR, and HER2 expression. 24.8% (546/2205) of patients were confirmed to regional LNM.

**Table 1:** Characteristics between the training set and the validation set

Characteristics	All Patients ***n*** = 2205 (%)	Training ***n*** = 1543 (%)	Validation ***n*** = 662 (%)	***P*** value
Age (years)				
Median	60	59	61	
≤ 60	1135 (51.5)	814 (52.8)	321 (48.5)	0.066
> 60	1070 (48.5)	729 (47.2)	341 (51.5)	
Race				
White	1691 (76.7)	1165 (75.5)	526 (79.5)	0.103
Black	364 (16.5)	271 (17.6)	93 (14.0)	
Others^a^	150 (6.8)	107 (6.9)	43 (6.5)	
Marital status				
Unmarried	959 (43.5)	670 (43.4)	289 (43.6)	0.750
Married	1173 (53.2)	819 (53.1)	354 (53.5)	
Unknown	73 (3.3)	54 (3.5)	19 (2.9)	
Grade				
I	103 (4.7)	67 (4.4)	36 (5.4)	0.666
II	299 (13.6)	207 (13.4)	92 (13.9)	
III	1701 (77.1)	1199 (77.7)	502 (75.8)	
IV	102 (4.6)	70 (4.5)	32 (4.9)	
Laterality				
Right	1129 (51.2)	777 (50.4)	352 (53.2)	0.225
Left	1076 (48.8)	766 (49.6)	310 (46.8)	
ER status				
Negative	1803 (81.8)	1265 (82.0)	538 (81.3)	0.691
Positive	402 (18.2)	278 (18.0)	124 (18.7)	
PR status				
Negative	1919 (87.0)	1342 (87.0)	577 (87.2)	0.905
Positive	286 (13.0)	201 (13.0)	85 (12.8)	
HER-2 status				
Negative	1091 (49.5)	751 (48.7)	340 (51.4)	0.155
Positive	76 (3.4)	60 (3.9)	16 (2.4)	
Unknown	1038 (47.1)	732 (47.4)	306 (46.2)	
Tumor size				
≤ 2 cm	572 (25.9)	399 (25.9)	173 (26.1)	0.976
> 2 cm and ≤ 5 cm	1157 (52.5)	812 (52.6)	345 (52.1)	
> 5 cm	476 (21.6)	332 (21.5)	144 (21.8)	
Regional lymph nodes				
Negative	1659 (75.2)	1164 (75.4)	495 (74.8)	0.741
Positive	546 (24.8)	379 (24.6)	167 (25.2)	

*ER* Estrogen receptor, *PR* Progesterone receptor, *HER-2* Human epidermal growth factor receptor-2

^a^Includes: American Indian, native Alaskan and Asian, Pacific Islander

Two thousand two hundred five MBC patients were stochastically divided into a training set (1543) and validation set (662). The regional LNM rate of the training set and the validation set was 24.6% (379/1543) and 25.2% (167/662), respectively. There were no significant differences in patient age, race, marital status, grade, laterality, receptor (ER, PR and HER-2) status, tumor size and regional lymph node status between the training set and the validation set (*P* > 0.05).

### Factors associated with regional LNM

The logistic regression model was established to evaluate the clinicopathological factors associated with regional LNM. Factors including age, race, marital status, grade, laterality, ER status, PR status, and tumor size were analyzed in binary logistic regression analysis. Univariate logistic regression analysis showed that unmarried, lower differentiation, positive expression of ER and tumors with a diameter > 2 cm were related to regional LNM. Multivariate logistic regression analysis further confirmed that poor differentiation (odds ratios (OR) = 2.69, 95% confidence interval (CI): 1.12–6.46, *P* = 0.026), undifferentiation (OR = 3.05, 95% CI: 1.10–8.45, *P* = 0.031), positive expression of ER (OR = 1.60, 95% CI: 1.14–2.23, *P* = 0.006), tumors with a diameter > 2 cm and ≤ 5 cm (OR = 2.19, 95% CI: 1.54–3.13, *P* < 0.001) and tumors with a diameter > 5 cm (OR = 5.51, 95% CI: 3.74–8.11, *P* < 0.001) were independent predictors of regional LNM in MBC patients (Table 2).

**Table 2:** Risk variables for regional lymph nodes metastasis determined by univariate and multivariate logistic regression analyses

Characteristics	Univariate analysis			Multivariate analysis		
	OR	***P*** value	95% CI	OR	***P*** value	95% CI
Age (years)						
≤ 60	Reference			Reference		
> 60	0.82	0.096	0.65–1.04	0.85	0.199	0.66–1.09
Race						
White	Reference			Reference		
Black	1.16	0.335	0.86–1.57	0.98	0.883	0.71–1.35
Others^a^	1.25	0.325	0.80–1.95	1.12	0.639	0.70–1.78
Marital status						
Unmarried	Reference			Reference		
Married	0.73	0.010	0.58–0.93	0.80	0.089	0.62–1.03
Unknown	0.83	0.560	0.43–1.58	0.95	0.875	0.48–1.87
Grade						
I	Reference			Reference		
II	2.07	0.119	0.83–5.16	1.83	0.208	0.71–4.68
III	3.65	0.003	1.56–8.54	2.69	0.026	1.12–6.46
IV	4.36	0.003	1.63–11.63	3.05	0.031	1.10–8.45
Laterality						
Right	Reference			Reference		
Left	0.82	0.094	0.65–1.04	0.79	0.058	0.62–1.01
ER status						
Negative	Reference			Reference		
Positive	1.49	0.007	1.12–1.98	1.60	0.006	1.14–2.23
PR status						
Negative	Reference			Reference		
Positive	1.08	0.644	0.77–1.52	0.97	0.870	0.65–1.44
Tumor size						
≤ 2 cm	Reference			Reference		
> 2 cm and ≤ 5 cm	2.39	< 0.001	1.68–3.39	2.19	< 0.001	1.54–3.13
> 5 cm	6.10	< 0.001	4.18–8.91	5.51	< 0.001	3.74–8.11

*OR* Odds ratios, *CI* Confidential interval, *ER* Estrogen receptor, *PR* progesterone receptor

^a^Includes: American Indian, native Alaskan and Asian, Pacific Islander

### Nomogram construction and validation

**Fig. 2:**
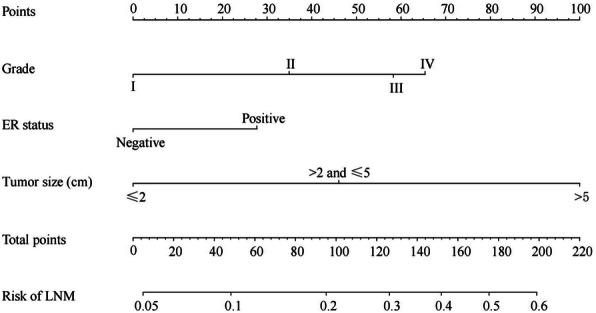
Nomogram predicting regional LNM in patients with MBC based on training cohort. The first row is the point assignment for each variable. Rows 2–4 indicate the variables included in the nomogram. For an individual patient, each variable is assigned a point value based on the histopathological characteristics. The points for each variable were summed and located on the total point line. And then, the bottom line shows the probability of the patient having regional LNM

A nomogram to predict preoperative regional LNM was established in the training set. Binary logistic regression analyses indicated that grade, ER status and tumor size were independent predictive factors of regional LNM in MBC patients. Therefore, we integrated those three variables to construct the nomogram (Fig. 2).

**Fig. 3:**
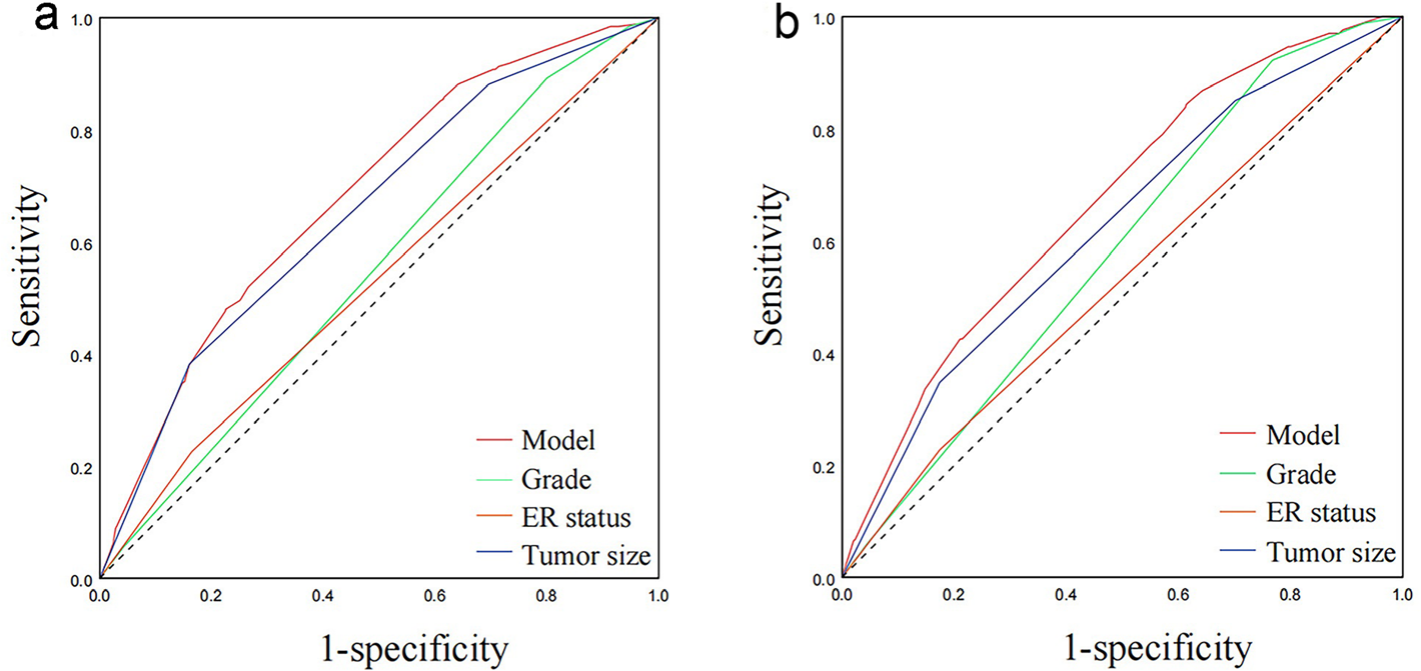
Receiver operating characteristics analyses of the nomogram of model and other predictors (grade, ER status and tumor size) based on the training (**a**) and validation (**b**) cohorts

**Fig. 4:**
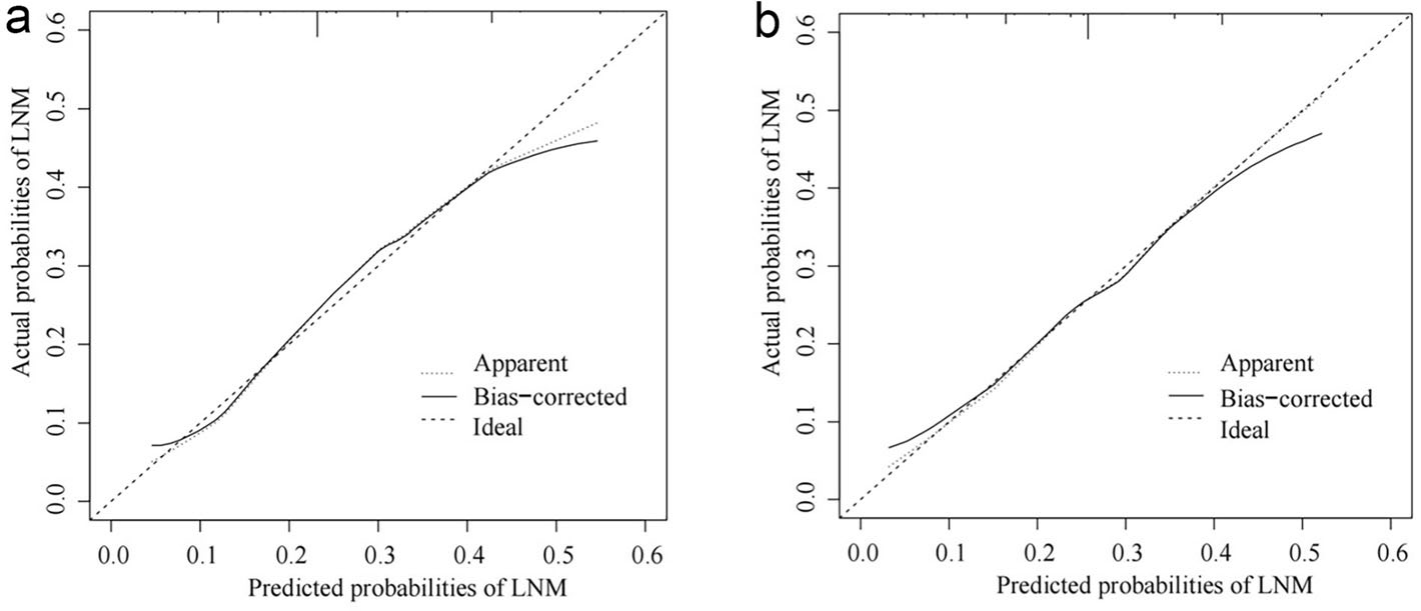
Internal (**a**) and external (**b**) calibration plots of the nomogram for predicting regional LNM in patients with MBC

The AUC in the training and validation set were 0.683 (95% CI: 0.653–0.713) and 0.667 (95% CI: 0.621–0.712) respectively, representing the moderate discrimination ability of the nomogram to estimate the status of regional LNM. More importantly, the AUC of all predictors alone were lower than the AUC of the nomogram, both in the training set (Fig. 3a) and validation set (Fig. 3b). Furthermore, the calibration plot with 1000 bootstrapping repetitions presented good agreement between the actual regional LNM and the predicted probability of regional LNM, no matter in the training set (Fig. 4a) and validation set (Fig. 4b).

### Clinical utility of the nomogram

**Fig. 5:**
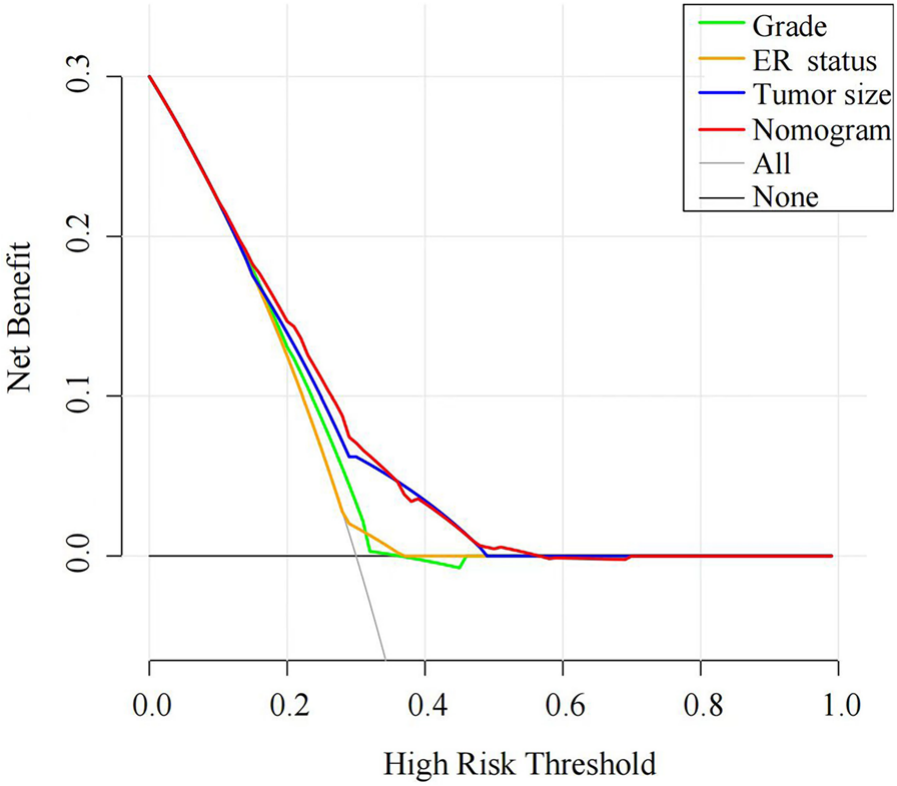
Decision curve for prediction of regional LNM for MBC. Black line: assume no patient will have regional LNM; gray line: assume all patients will have regional LNM; orange line: binary decision rule based on ER status alone; green line: binary decision rule based on grade alone; blue line: binary decision rule based on tumor size alone; red line: decision based on nomogram. The x-axis and the y-axis were the threshold probability and the net benefit, respectively

DCA was performed to evaluate the clinical utility of the nomogram based on net benefits at different threshold probabilities. Compared with grade, ER status and tumor size, the increased net benefit of the nomogram was the largest, which indicated that the nomogram was a reliable clinical tool for predicting regional LNM in MBC patients (Fig. 5).

## Discussion

MBC is a rare histological subtype of BC, most of which commonly present with a triple negative phenotype [34, 35]. Compared with invasive ductal carcinoma, MBC is characterized by lower differentiation, larger tumor size, less LNM and poorer clinical outcomes [36, 37]. In this study, among all 2205 patients, 1803 (81.8%) were poorly differentiated or undifferentiated, 402 (18.2%) were ER-positive, 286 (13.0%) were PR-positive, 76 (6.5%) were HER-2-positive, 1633 (74.1%) had tumors greater than 2 cm, 546 (24.8%) observed LNM. These results are consistent with the findings of previous studies.

LNM is considered a significant negative prognostic factor and is vitally important for therapeutic decision-making for MBC patients. But, current preoperative imaging modalities do not have high sensitivities and specificities in the diagnosis of LNM. Moreover, MBC has a wide range of histological patterns and extremely minimal metaplastic area, so it is difficult to identify by fine-needle aspiration or core biopsy before operation [38]. Leyrer CM et al. reported only 41% (46/113) patients were identified preoperatively as MBC on initial image-guided core biopsy [31]. Furthermore, nomogram, as a simple and advanced prediction tool, can estimate individualized risk by integrating substantial clinicopathological characteristics [39]. Therefore, it is necessary to establish a simple and sensitive preoperative prediction model of regional LNM in MBC patients.

In our study, grade, ER status and tumor size were considered independent predictors for regional LNM in patients with MBC. Then, those three clinicopathological variables were incorporated into a preoperative estimation model of regional LNM risk. To the best of our knowledge, this is the first population-based study to develop and validate a nomogram for predicting the preoperative individualized risk of regional LNM in MBC patients. In both training set and validation set, the AUC of the nomogram was higher than 0.6 and the calibration curves corresponded with the idealized 45° line, which demonstrated excellent discrimination and calibration of the nomogram. Nevertheless, great discrimination and calibration of the nomogram are not sufficient because they do not equal to clinical utility. In addition, MBC has a low incidence rate, and most available studies are retrospective studies of small samples. Hence, we used DCA to estimate the clinical usefulness, and DCA curves revealed greater net benefit of the established nomogram model. In other words, through this nomogram, we can accurately predict the regional lymph node status of MBC patients.

ER status as one of the most influential independent predictors of LNM has been reported in some studies. Gann PH et al. studied data from 18,025 breast carcinoma cases and suggested tumors lacking ER had a significantly lower risk of LNM than tumors containing ER [40]. Additionally, Ye FG et al. revealed ER status was an independently associated with a higher likelihood of LNM (OR = 5.254, 95% CI: 0.392–19.834, *P* < 0.014) [41]. Compared with the previous studies, we achieved a consistent conclusion. In our study, 30.9% of women with ER positive cancers were found to have positive regional lymph nodes compared to 23.2% of women with ER negative cancers (*P* = 0.006).

Several studies demonstrated that histologic grade was related to LNM status. Kollias J et al. retrospectively analyzed the medical records of 2684 BC patients, and showed that 29% of the patients with grade III cancers had positive lymph nodes, while the proportion of positive lymph nodes with grade I and grade II was 11 and 18%, respectively (*P* = 0.006) [42]. Besides, Bruno C et al. revealed the high pathological grade indicated high axillary nodal involvement in BC. The risk of axillary nodal involvement in grade III tumors doubled compared to grade I tumors (37.8% versus 18.3%) [43]. In our study, the highest positive rate of regional lymph nodes was observed in grade III MBC, which is consistent with the above conclusion.

The relationship between tumor size and LNM in BC patients have been widely reported in previous researches. In 1989, Carter et al. showed the incidence of LNM was approximately 31.1% (2591/8319) in the BC patients with tumors less than 2 cm, while the proportion of LNM was as high as 70.0% (1889/2698) in the BC patients with tumors 5 cm or greater [44]. Moreover, in 2006, Wada et al. reported nearly 50% (62/116) of the BC patients with T2 tumor (> 2.0 cm) had positive non-sentinel lymph nodes [45]. Similarly, our study found that tumor size was an independent risk factor significantly associated with LNM in MBC patients. In our data, the percentages of regional lymph node positive tumors with greatest dimensions less than or equal to 2 cm and greater than 5 cm were 12.2% (70/572) and 42.6% (203/476).

We successfully constructed a nomogram based on a large population-based cohort for assessing the potential risk of regional LNM in MBC patients by utilizing grade, ER status, and tumor size. The established predictive model exhibited excellent performance, and was based on easily available clinicopathological factors. Therefore, the preoperative prediction of regional LNM could be accurately and conveniently identified on the nomogram by collecting the readily accessible information.

Although the nomogram had good accuracy for LNM prediction in MBC patients, some potential limitations of our study should be noted. First, due to the nature of retrospective analyses, we could not exclude selection bias. For example, some patients were excluded due to missing data, which may cause selection bias. Second, Ki-67 has been identified as an independent predictor of LNM in BC [46]. Unfortunately, it was not recorded in the SEER database. Thus we cannot incorporate this important factor into our nomogram. Finally, both training and validation sets came from the SEER database, which may lead to overfitting the model. The nomogram needs to be validated in more external force columns from other institutions to demonstrate its reproducibility.

## Conclusion

In conclusion, through logistic regression analysis, we found that lower differentiation, ER positive status and larger tumor size were independent risk factors associated with regional LNM in MBC. Based on these clinical risk factors, we established the first nomogram that could accurately and easily predict the preoperative individualized risk of regional LNM for MBC patients, thus contributing to treatment decision making.

## Data Availability

The datasets generated and/or analyzed during the current study are available in the SEER database (https://seer.cancer.gov/).

## Abbreviations

MBC: Metaplastic breast cancer
LNM: Lymph nodes metastasis
SEER: Surveillance, Epidemiology, and End Results
ROC: Receiver operating characteristic
AUC: Area under the curve
DCA: Decision curve analysis
ER: Estrogen receptor
PR: Progesterone receptor
HER-2: Human epidermal growth factor receptor-2
CI: Confidential interval
OR: Odds ratios

## References

[1] Pezzi CM, Patel-Parekh L, Cole K, Franko J, Klimberg VS, Bland K (2007). Characteristics and treatment of metaplastic breast cancer: analysis of 892 cases from the National Cancer Data Base. Ann Surg Oncol.

[2] Al Sayed AD, El Weshi AN, Tulbah AM, Rahal MM, Ezzat AA (2006). Metaplastic carcinoma of the breast clinical presentation, treatment results and prognostic factors. Acta Oncol.

[3] Toumi Z, Bullen C, Tang AC, Dalal N, Ellenbogen S (2011). Metaplastic breast carcinoma: a case report and systematic review of the literature. Pathol Int.

[4] Gibson GR, Qian D, Ku JK, Lai LL (2005). Metaplastic breast cancer: clinical features and outcomes. Am Surg.

[5] Huvos AG, Lucas JC, Foote FW (1973). Metaplastic breast carcinoma. Rare form of mammary cancer. N Y State J Med.

[6] Zdenkowski N, Butow P, Spillane A, Douglas C, Snook K, Jones M, Oldmeadow C, Fewster S, Beckmore C, Boyle FM, for the Australia and New Zealand Breast Cancer Trials Group (2018). Single-arm longitudinal study to evaluate a decision aid for women offered neoadjuvant systemic therapy for operable breast cancer. J Natl Compr Cancer Netw.

[7] Recht A, Comen EA, Fine RE, Fleming GF, Hardenbergh PH, Ho AY, Hudis CA, Hwang ES, Kirshner JJ, Morrow M, Salerno KE, Sledge GW, Solin LJ, Spears PA, Whelan TJ, Somerfield MR, Edge SB (2016). Postmastectomy radiotherapy: an American Society of Clinical Oncology, American Society for Radiation Oncology, and Society of Surgical Oncology focused guideline update. J Clin Oncol.

[8] Frasier LL, Holden S, Holden T, Schumacher JR, Leverson G, Anderson B, Greenberg CC, Neuman HB (2016). Temporal trends in postmastectomy radiation therapy and breast reconstruction associated with changes in National Comprehensive Cancer Network guidelines. JAMA Oncol.

[9] Wargotz ES, Norris HJ (1991). Metaplastic carcinomas and sarcomas of the breast. Am J Clin Pathol.

[10] Chen W, Wang C, Fu F, Yang B, Chen C, Sun Y (2020). A model to predict the risk of lymph node metastasis in breast cancer based on clinicopathological characteristics. Cancer Manag Res.

[11] Barone JE, Tucker JB, Perez JM, Odom SR, Ghevariya V (2005). Evidence-based medicine applied to sentinel lymph node biopsy in patients with breast cancer. Am Surg.

[12] Tan W, Xie X, Huang Z, Chen L, Tang W, Zhu R, Ye X, Zhang X, Pan L, Gao J, Tang H, Zheng W (2020). Construction of an immune-related genes nomogram for the preoperative prediction of axillary lymph node metastasis in triple-negative breast cancer. Artif Cells Nanomed Biotechnol.

[13] DeSantis CE, Ma J, Gaudet MM, Newman LA, Miller KD, Goding Sauer A, Jemal A, Siegel RL (2019). Breast cancer statistics, 2019. CA Cancer J Clin.

[14] de Sire A, Losco L, Cigna E, Lippi L, Gimigliano F, Gennari A, Cisari C, Chen HC, Fusco N, Invernizzi M (2020). Three-dimensional laser scanning as a reliable and reproducible diagnostic tool in breast cancer related lymphedema rehabilitation: a proof-of-principle study. Eur Rev Med Pharmacol Sci.

[15] de Sire A, Invernizzi M, Lippi L, Cisari C, Özçakar L, Franchignoni F (2020). Blurred lines between axillary web syndrome and Mondor’s disease after breast cancer surgery: a case report. Ann Phys Rehabil Med.

[16] Giuliano AE, Ballman KV, McCall L, Beitsch PD, Brennan MB, Kelemen PR, Ollila DW, Hansen NM, Whitworth PW, Blumencranz PW, Leitch AM, Saha S, Hunt KK, Morrow M (2017). Effect of axillary dissection vs no axillary dissection on 10-year overall survival among women with invasive breast cancer and sentinel node metastasis: the ACOSOG Z0011 (Alliance) randomized clinical trial. JAMA.

[17] Paolucci T, Bernetti A, Bai AV, Segatori L, Monti M, Maggi G (2021). The sequelae of mastectomy and quadrantectomy with respect to the reaching movement in breast cancer survivors: evidence for an integrated rehabilitation protocol during oncological care. Support Care Cancer.

[18] Paolucci T, Bernetti A, Paoloni M, Capobianco SV, Bai AV, Lai C, Pierro L, Rotundi M, Damiani C, Santilli V, Agostini F, Mangone M (2019). Therapeutic alliance in a single versus group rehabilitative setting after breast cancer surgery: psychological profile and performance rehabilitation. Biores Open Access.

[19] Stubblefield MD (2017). The underutilization of rehabilitation to treat physical impairments in breast cancer survivors. PM R.

[20] Invernizzi M, de Sire A, Lippi L, Venetis K, Sajjadi E, Gimigliano F, Gennari A, Criscitiello C, Cisari C, Fusco N (2020). Impact of rehabilitation on breast cancer related fatigue: a pilot study. Front Oncol.

[21] Yang S, Chu S, Gao Y, Ai Q, Liu Y (2019). A narrative review of cancer-related fatigue (CRF) and its possible pathogenesis. Cells.

[22] Michelotti A, Invernizzi M, Lopez G, Lorenzini D, Nesa F, De Sire A (2019). Tackling the diversity of breast cancer related lymphedema: perspectives on diagnosis, risk assessment, and clinical management. Breast.

[23] Abrahams HJG, Gielissen MFM, Schmits IC, Verhagen C, Rovers MM, Knoop H (2016). Risk factors, prevalence, and course of severe fatigue after breast cancer treatment: a meta-analysis involving 12 327 breast cancer survivors. Ann Oncol.

[24] Fernández de Larrea-Baz N, Pérez-Gómez B (2020). Primary breast cancer and health related quality of life in Spanish women: the EpiGEICAM case-control study. Sci Rep.

[25] El Haidari R, Abbas LA (2020). Factors associated with health-related quality of life in women with breast cancer in the Middle East: a systematic review. Cancers (Basel).

[26] Cho N, Han W, Han BK, Bae MS, Ko ES, Nam SJ, Chae EY, Lee JW, Kim SH, Kang BJ, Song BJ, Kim EK, Moon HJ, Kim SI, Kim SM, Kang E, Choi Y, Kim HH, Moon WK (2017). Breast cancer screening with mammography plus ultrasonography or magnetic resonance imaging in women 50 years or younger at diagnosis and treated with breast conservation therapy. JAMA Oncol.

[27] Shieh Y, Eklund M, Madlensky L, Sawyer SD, Thompson CK, Stover Fiscalini A, et al. Breast cancer screening in the precision medicine era: risk-based screening in a population-based trial. J Natl Cancer Inst. 2017;109(5). 10.1093/jnci/djw290. 10.1093/jnci/djw290 28130475

[28] Lee CH, Dershaw DD, Kopans D, Evans P, Monsees B, Monticciolo D, Brenner RJ, Bassett L, Berg W, Feig S, Hendrick E, Mendelson E, D'Orsi C, Sickles E, Burhenne LW (2010). Breast cancer screening with imaging: recommendations from the Society of Breast Imaging and the ACR on the use of mammography, breast MRI, breast ultrasound, and other technologies for the detection of clinically occult breast cancer. J Am Coll Radiol.

[29] Friedewald SM, Rafferty EA, Rose SL, Durand MA, Plecha DM, Greenberg JS, Hayes MK, Copit DS, Carlson KL, Cink TM, Barke LD, Greer LN, Miller DP, Conant EF (2014). Breast cancer screening using tomosynthesis in combination with digital mammography. JAMA.

[30] Ribeiro-Silva A, Luzzatto F, Chang D, Zucoloto S (2001). Limitations of fine-needle aspiration cytology to diagnose metaplastic carcinoma of the breast. Pathol Oncol Res.

[31] Leyrer CM, Berriochoa CA, Agrawal S, Donaldson A, Calhoun BC, Shah C, Stewart R, Moore HCF, Tendulkar RD (2017). Predictive factors on outcomes in metaplastic breast cancer. Breast Cancer Res Treat.

[32] Li Y, Chen M, Pardini B, Dragomir MP (2019). The role of radiotherapy in metaplastic breast cancer: a propensity score-matched analysis of the SEER database. J Transl Med.

[33] Schroeder MC, Rastogi P, Geyer CE, Miller LD, Thomas A (2018). Early and locally advanced metaplastic breast cancer: presentation and survival by receptor status in surveillance, epidemiology, and end results (SEER) 2010–2014. Oncologist.

[34] Weigelt B, Kreike B, Reis-Filho JS (2009). Metaplastic breast carcinomas are basal-like breast cancers: a genomic profiling analysis. Breast Cancer Res Treat.

[35] Weigelt B, Ng CK, Shen R, Popova T, Schizas M, Natrajan R (2015). Metaplastic breast carcinomas display genomic and transcriptomic heterogeneity [corrected]. Mod Pathol.

[36] Park HS, Park S, Kim JH, Lee JH, Choi SY, Park BW, Lee KS (2010). Clinicopathologic features and outcomes of metaplastic breast carcinoma: comparison with invasive ductal carcinoma of the breast. Yonsei Med J.

[37] Dieci MV, Orvieto E, Dominici M, Conte P, Guarneri V (2014). Rare breast cancer subtypes: histological, molecular, and clinical peculiarities. Oncologist.

[38] Beatty JD, Atwood M, Tickman R, Reiner M (2006). Metaplastic breast cancer: clinical significance. Am J Surg.

[39] Lu YJ, Yang Y, Yuan YH, Wang WJ, Cui MT, Tang HY, Duan WM (2020). A novel nomogram based on SEER database for the prediction of liver metastasis in patients with small-cell lung cancer. Ann Palliat Med.

[40] Gann PH, Colilla SA, Gapstur SM, Winchester DJ, Winchester DP (1999). Factors associated with axillary lymph node metastasis from breast carcinoma: descriptive and predictive analyses. Cancer.

[41] Ye FG, Xia C, Ma D, Lin PY, Hu X, Shao ZM (2018). Nomogram for predicting preoperative lymph node involvement in patients with invasive micropapillary carcinoma of breast: a SEER population-based study. BMC Cancer.

[42] Kollias J, Murphy CA, Elston CW, Ellis IO, Robertson JF, Blamey RW (1999). The prognosis of small primary breast cancers. Eur J Cancer.

[43] Cutuli B, Velten M, Martin C (2001). Assessment of axillary lymph node involvement in small breast cancer: analysis of 893 cases. Clin Breast Cancer.

[44] Carter CL, Allen C, Henson DE (1989). Relation of tumor size, lymph node status, and survival in 24,740 breast cancer cases. Cancer.

[45] Wada N, Imoto S, Yamauchi C, Hasebe T, Ochiai A (2006). Predictors of tumour involvement in remaining axillary lymph nodes of breast cancer patients with positive sentinel lymph node. Eur J Surg Oncol.

[46] Orsaria P, Caredda E, Genova F, Materazzo M, Capuano I, Vanni G, Granai AV, de Majo A, Portarena I, Sileri P, Petrella G, Palombi L, Buonomo OC (2018). Additional nodal disease prediction in breast cancer with sentinel lymph node metastasis based on clinicopathological features. Anticancer Res.

